# Selective costimulation by IL-15R/IL-15, but not IL-2R/IL-2, allows the induction of high numbers of tumor-specific CD8+ T cells by human dendritic cells matured in conditions of acute inflammation

**DOI:** 10.1186/2051-1426-3-S2-P223

**Published:** 2015-11-04

**Authors:** Morten Hansen, Eva Wieckowski, Inge Marie Svane, Robert P Edwards, Pawel Kalinski

**Affiliations:** 1 Copenhagen University Hospital, Herlev, Denmark; 2 University of Pittsburgh, Pittsburgh, PA, USA; 3 Center for Cancer Immune Therapy, Herlev Hospital, Copenhagen University, Herlev, Denmark

## 

Conventional dendritic cells (DC) are believed to rely on membrane-bound IL-2Rα to trans-present soluble IL-2 and costimulate T cell activation and expansion. In contrast, Langerhans cells have been shown to use membrane-bound IL-15Rα/IL-15 complex to activate T cells. Here we show that, while the expansion of tumor-specific CD8^+^ T cells by DC matured in the presence of chronic inflammatory mediators (PGE_2_, TNFα IL-1β, IL-6) fully depends on expression of IL-2Rα, CD8^+^ T cell expansion induced by IL-12p70-producing DC matured by interferon's and Toll-Like receptor ligands (type-1-polarized; DC1) is both more effective and independent of IL-2Rα expression. While DC1-expressed IL-15Rα promotes the expansion of tetramer-specific CD8^+^ T cells, their secreted levels of IL-12p70 determines the degree of CD8^+^ T cell functionality as evidenced by tumor antigen-specific release of IFNγ and TNFα. In accordance with the in vivo advantage of utilizing an IL-2-independent pathway of costimulation of tumor-specific T cells, in a retrospectively analyzed cohort of patients with metastatic malignant melanoma treated with cyclophosphamide and tumor-antigen transfected DCs (NCT00978913) we observed a highly significant inverse relation between overall survival and expression of IL-2Rα on DC vaccine products (p = 0.009). The differential usage of IL-2Rα/IL-2 versus IL-15Rα/IL-15 pathways by subsets of DCs helps to explain the role of different types of inflammation in memory formation, exhaustion of CD8^+^ T cell responses and progression of cancer. Furthermore, *ex vivo* induction of IL-15Rα/IL-15 dependent signaling might improve adoptive T cell therapies targeting tumors with well-defined and undefined tumor rejection antigens.

